# Association of Dietary Vitamin D Intake, Serum 25(OH)D_3_, 25(OH)D_2_ with Cognitive Performance in the Elderly

**DOI:** 10.3390/nu13093089

**Published:** 2021-09-02

**Authors:** RuTong Wang, Weijing Wang, Ping Hu, Ronghui Zhang, Xue Dong, Dongfeng Zhang

**Affiliations:** 1 Department of Epidemiology and Health Statistics, the School of Public Health of Qingdao University, 308 Ningxia Road, Qingdao 266071, China; rt9707vicky@126.com (R.W.); wangwj793@126.com (W.W.); zhangrh9634@163.com (R.Z.); dongxue199411@126.com (X.D.); 2 Qingdao Municipal Center for Disease Control and Prevention, Qingdao Institute of Preventive Medicine, Qingdao 266033, China; hupingqu@163.com

**Keywords:** cognitive performance, dietary vitamin D, 25-hydroxyvitamin D_3_, 25-hydroxyvitamin D_2_, does–response

## Abstract

Background: As life expectancy increases, cognitive performance decline in the elderly has become one of the major global challenges. We aimed to evaluate the association of dietary vitamin D (VD), serum 25-hydroxyvitamin D3 (25(OH)D_3_), 25-hydroxyvitamin D2 (25(OH)D_2_), and total 25-hydroxyvitamin (25(OH)D) concentration with cognitive performance in older Americans. Methods: The data from the National Health and Nutrition Examination Survey (NHANES), 2011–2014 was used. The cognitive performance was assessed by the Consortium to Establish a Registry for Alzheimer’s Disease (CERAD) Word Learning sub-test, Animal Fluency test, and Digit Symbol Substitution Test (DSST). A binary logistic regression model was applied to evaluate the association between VD and cognitive performance, and restricted cubic spline model was adopted to evaluate the dose–response relationship. Results: While comparing to the lowest dietary VD intake group, the multivariate adjusted odds ratios (ORs) and 95% confidence intervals (CIs) of the highest dietary VD intake group were 0.51 (0.36–0.72) for the Animal Fluency test score and 0.45 (0.31–0.66) for DSST score, respectively; and those of serum total 25(OH)D and 25(OH)D_3_ concentration were 0.68 (0.47–0.97) and 0.62 (0.44–0.86) for DSST score. L-shaped relationships were identified for dietary VD intake, serum total 25(OH)D and 25(OH)D_3_ concentration with cognition performance. The associations between dietary VD intake, serum total 25(OH)D and cognitive performance were non-significant when stratified by gender. Conclusions: The study indicates that dietary VD intake, serum total 25(OH)D and 25(OH)D_3_ concentration were positively associated with cognitive performance. Further studies are needed to clarify the possible effects of dietary VD intake and serum 25(OH)D_2_, 25(OH)D_3_ on cognitive performance.

## 1. Introduction

As life expectancy increases, cognitive performance decline in the elderly has become one of the major global challenges [1]. According to projections, the number of people worldwide living with dementia will rise to an estimated 152 million by 2050 [2]. Considering the social and economic burden of cognitive decline especially dementia, it is particularly important to control, delay, and prevent cognitive decline. Genetic factors, the history of illness, and life stress may increase the risk of cognitive performance decline. Additionally, healthy dietary habits, such as proper intake of vitamin C, vitamin E, and polyunsaturated fatty acids, may have a protective effect on cognitive performance [3,4,5].

Vitamin D (VD), as one of the common fat-soluble vitamins, plays an important role not only in bone growth and development, but also in cell differentiation and the immune system [6]. VD is neuroprotective, anti-inflammatory, and antioxidant, so it is considered to be one of the protective factors for cognitive performance [7]. To assess the vitamin D status, the dietary VD intake and serum 25-hydroxyvitamin D (25(OH)D) concentration were commonly used [8]. Dietary intake is a source of VD [9], but the association between dietary VD intake and cognition remains controversial [8,10,11,12]. A cross-sectional study in Dutch and a follow-up study in men showed that dietary VD intake was not associated with cognitive performance [8,11]. Other studies suggested an association between dietary VD intake and cognitive performance [10,12]. Moreover, to our knowledge, the dose–response relationship between intake of dietary VD and cognitive performance has not yet been explored.

In addition, the effect of serum 25(OH)D on cognitive performance is still controversial [8,13,14,15,16,17,18]. A recent cohort in Boston-area Puerto Ricans showed that the association of serum 25(OH)D concentration with individual cognitive test scores was not statistically significant [19]. However, another recent study in women suggested that higher 25(OH)D concentrations might have negative cognitive effects [20]. Additionally, currently, no study investigated the dose–response relationships between them. Due to different chemical construction and physiological activities of 25-hydroxyvitamin D3 (25(OH)D_3_), D2 (25(OH)D_2_) in serum [21,22,23], the effect of serum 25(OH)D_2_, 25(OH)D_3_ on cognition might differ. However, these associations have not been extensively explored up until now.

Therefore, we aimed to investigate the association and dose–response relationships of dietary VD intake, serum total 25(OH)D, serum 25(OH)D_2_, and serum 25(OH)D_3_ with cognitive performance in older Americans based on the data from the National Health and Nutrition Examination Survey (NHANES).

## 2. Materials and Methods

### 2.1. Study Population

**Figure 1:**
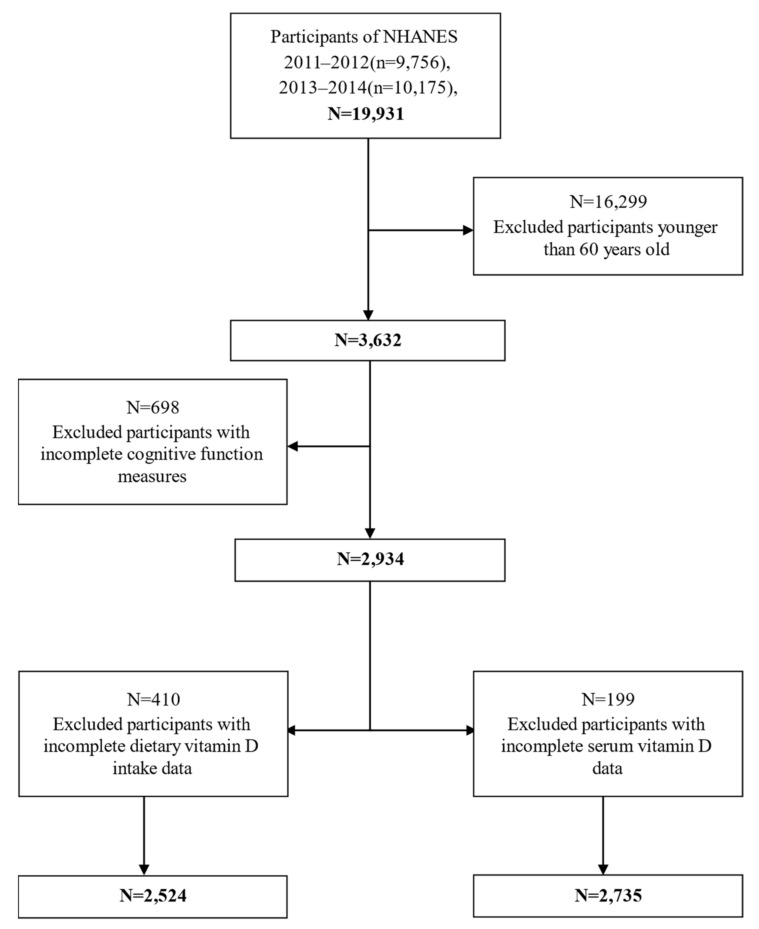
Flow chart of the screening process for the selection of eligible dietary VD intake and serum 25(OH)D participants.

The data on dietary VD intake, serum 25(OH)D concentration, and cognitive performance test scores were obtained from the two cycles of NHANES: 2011–2012 and 2013–2014. Since only one-third of NHANES samples were serologically tested each year, we will waste a deal of valuable information when excluding the participants with both dietary VD intake and serum 25(OH)D simultaneously. Thus, we chose two different samples to study the association between dietary VD intake, serum 25(OH)D and cognition, respectively. A total of 19,931 Americans were included in the study, leaving 3632 older participants after excluding those under 60 years old. Among them, we retained 2934 participants with complete cognitive assessment test scores. Additionally, we separately excluded participants with incomplete dietary VD intake data (n = 410) and serum VD data (n = 199). Finally, a total of 2425 survey participants for dietary VD intake and a total of 2735 examination participants for serum 25(OH)D were included in this study (Figure 1).

### 2.2. Cognitive Performance

The NHANES database contained cognitive performance data that were obtained through the Consortium to Establish a Registry for Alzheimer’s Disease (CERAD) Word Learning sub-test (assessing the ability to learn new verbal information), the Animal Fluency test (examining categorical verbal fluency in executive function) and the Digit Symbol Substitution Test (DSST) (assessing processing speed, sustained attention and working memory).

The CERAD test included three immediate recall tests and one delayed recall test. In three immediate recall tests, participants read 10 unrelated words and then recalled as many words as possible immediately. Delayed recall test was completed after Animal Fluency test and DSST. The maximum score of each test was 10. The total score of CERAD test was the sum of three immediate recall tests and one delayed recall test. The Animal Fluency test participants were renamed as many animals as possible within one minute. The score was the sum of the correct answers. The DSST asked participants to copy the corresponding symbols from 133 boxes within two minutes. The score ranges from 0 to 133, which was the sum of the number of correct matches [24]. Higher scores of three tests indicated better cognitive performance.

As the CERAD test, Animal Fluency test, and DSST lacked recognized standards for defining low cognitive performance, the study referred to the processing methods in the relevant published research [25]. Additionally, the minimum quartile of these three test scores was used as the cutoff point. Furthermore, considering that the participants were aged over 60 years old and the effect of age on cognitive performance was even more significant, the three test scores were further adjusted according to age (≥60 years and ≥70 years) [26], and these cutoff points are shown in Table 1. Participants with scores lower than or equal to cutoff points were considered to have low cognitive performance, while those with scores greater than cutoff points were considered to have normal cognitive performance.

### 2.3. The Intake of Dietary VD

The intake of dietary VD was assessed by the two 24 h diet recall interviews in NHANES. The details of NHANES dietary survey were described elsewhere [27]. The collected data of dietary VD intake was further processed and analyzed. Referred to dietary VD Recommended Nutrient Intakes (RNIs) from the Food and Agriculture Organization of the United Nations (FAO) and World Health Organization (WHO) (5.00 μg/d) [28], participants with dietary VD intake lower than RNIs were classified as a reference group (Q1: ≤5.00 μg/d). For participants with dietary VD intake higher than RNIs, we used two methods to bring them into the model: (a) participants being evenly divided into two groups (Q2: ≤20.75 μg/d; Q3: >20.75 μg/d); (b) participants being classified as one group (Q2: >5.00 μg/d).

### 2.4. Laboratory Measurement of 25(OH)D

Participants had to fast for nine hours before drawing blood, which was then processed into vials and stored in the Mobile Examination Center (MEC). The vials were then refrigerated or frozen and shipped to laboratories around the United States of America [29,30]. High-performance liquid chromatography (HPLC) tandem mass spectrometry was used to analyzed serum 25(OH)D_3_ and 25(OH)D_2_ concentration (nmol/L), and the sum of them was the serum total 25(OH)D concentration (nmol/L). NHANES proposed that when the concentrations of 25(OH)D_2_ were lower than the limit of detection (LOD), the 25(OH)D_2_ concentration was shown as the imputed value (1.45 nmol/L). [31]. The optimal level of 25(OH)D has been debated [32,33,34]; thus, participants were divided into three groups according to the tertiles of serum total 25(OH)D and 25(OH)D_3_ concentration (nmol/L). Additionally, participants were divided into two groups based on the 25(OH)D_2_ concentration imputed value (Q1: ≤1.45 nmol/L and Q2: >1.45 nmol/L).

### 2.5. Covariates

When referring to previous research [4,35,36], we included a number of factors (age, gender, race, marital status, educational level, poverty-income ratio, body mass index, smoking and drinking status, hypertension, diabetes, and stroke) as covariates. Given the influence of seasons and physical activities on dietary VD intake and serum 25(OH)D concentration, we also included the seasons of exam and physical activity level as covariates. In addition, the total energy intake was also taken as a covariate for dietary VD intake. Hypertension and diabetes were defined by a combination of the physician’s history of diagnosis in the questionnaire and laboratory measurements of systolic/diastolic blood pressure and glycated hemoglobin.

### 2.6. Statistical Analysis

We referred to the NHANES weight analysis guide [37] to process the new sample weights after combining two cycles. Kolmogorov–Smirnov normality test was used to test the normality of continuous variables. We used the mean ± standard deviation (SD) to describe normally distributed variables and the median (standard error) to describe non-normally distributed variables. If the variable was normally distributed, the Student *t*-test was used to compare the mean levels between the low cognitive performance group and the normal cognitive performance group. If the variable was not normally distributed, Mann–Whitney U test was used. Chi-square test was selected to compare the percentage of categorical variables between different groups.

Binary logistic regression analyses were used to explore whether there were associations between dietary VD intake, serum total 25(OH)D, 25(OH)D_2_, and 25(OH)D_3_ and cognitive performance. Age and gender were adjusted for in model 1, and the other covariates were further adjusted for in model 2. We also conducted a gender stratified analysis. In addition, we used restricted cubic spline to further explore the dose–response relationships between serum 25(OH)D, dietary VD intake and different cognitive performance test scores, which located 5 percent, 50 percent, and 95 percent of the exposure distribution in the logistic regression after adjusting all covariates. The size of a test was 0.05, and the results were considered statistically significant when the bilateral *p*-value ≤ 0.05.

## 3. Results

### 3.1. Sample Characteristics

The characteristics of participants for dietary VD intake survey by cognitive performance are shown in Table 2. We could see that people who were non-Hispanic white, with lower educational level, lower income, higher prevalence of diabetes, hypertension, and stroke, often alcohol drinkers, with lower physical activity level, and higher total energy intake had lower cognitive performance. Additionally, the characteristics of participants for serum 25(OH)D by cognitive performance are shown in Table 3. We could see that people who were non-Hispanic white, with lower educational level, often drink alcohol, have lower physical activity level, higher prevalence of diabetes, hypertension, and stroke had lower cognitive performance. We included the interaction term of exam season and serum 25(OH)D multiplication in the multi-factor logistics regression model, and interaction terms were not statistical significance in the cognitive tests of CERAD, Animal Fluency test, and DSST.

### 3.2. Association between Dietary VD and Cognition

While comparing to the lowest group of dietary VD intake, the multivariate adjusted ORs (95% CIs) of the highest group of dietary VD intake was 0.51 (0.36–0.72) for the Animal Fluency test score and 0.45 (0.31–0.66) for the DSST score, respectively. After only adjusting for age and gender, dietary VD intake higher than 20.75 μg/d was associated with a reduced low cognitive performance risk assessed by CERAD. When the dietary VD intake was divided into two groups by RNIs, the associations persisted even after adjusting all covariates with ORs (95% CIs) being 0.60 (0.41–0.89) for the Animal Fluency test score and 0.59 (0.40–0.86) for DSST score (Table 4).

Model 2 showed that when the dietary VD intake was higher than 20.75 μg/d, it had statistically significant effects on cognitive performance assessed by DSST in both males (0.46 (0.26–0.82)) and females (0.50 (0.30–0.83)). In terms of the Animal Fluence test, the fully adjusted model was statistically significant (0.38 (0.22–0.67)) when the dietary VD intake higher than 20.75 μg/d in males only. Moreover, when the dietary VD intake was divided into two groups by RNIs, the associations between the VD and Animal Fluency test score (0.68 (0.47–1.00)) and DSST score (0.53 (0.31–0.93)) were more pronounced in female whose dietary VD intake higher than 5.00 μg/d (Table 5).

### 3.3. Association between Serum Total 25(OH)D and Cognition

When the serum total 25(OH)D concentration was higher than 86.30 nmol/L, the serum total 25(OH)D was positively correlated with DSST score (0.68 (0.47–0.97)) (Table 5). After adjusting for all covariates, the effect of serum total 25(OH)D concentration on cognitive performance was not significantly different in males and females. When the total 25(OH)D concentration was higher than 61.41 nmol/L, the association between serum total 25(OH)D concentration and Animal Fluency test score was statistically significant in males, but not in females (Model 1). Additionally, the association between the serum total 25(OH)D concentration and CERAD score was only statistically significant in the male when total 25(OH)D concentration was higher than 86.30 nmol/L in Model 1 (Table 6).

### 3.4. Association between Serum 25(OH)D_2_, 25(OH)D_3_ and Cognition

In model 1, when the 25(OH)D_3_ concentration was higher than 55.14 nmol/L, the serum 25(OH)D_3_ concentration was associated with the Animal Fluency test score and DSST score. In the fully adjusted model, the serum 25(OH)D_3_ concentration was associated with DSST score (0.62 (0.44–0.86)) when the serum 25(OH)D_3_ concentration was higher than 80.63 nmol/L. However, this association was not found between serum 25(OH)D_2_ and cognitive test scores (Table 6).

### 3.5. Dose–Response Relationships

**Figure 2:**
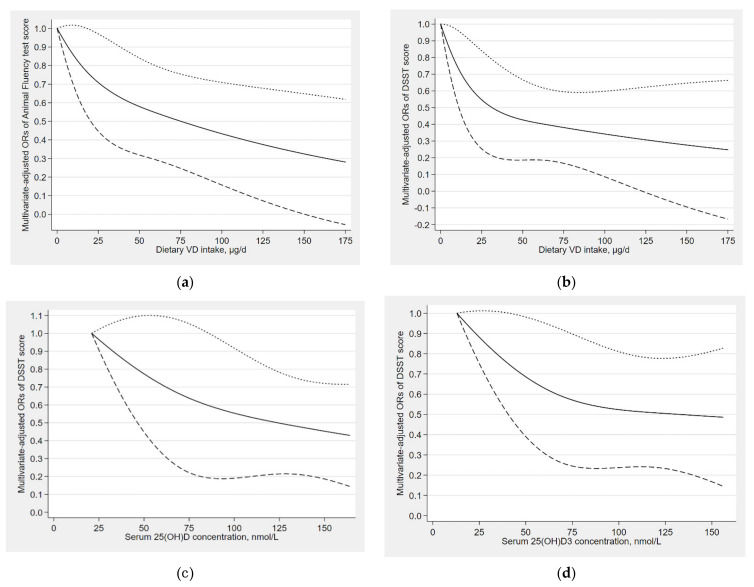
(**a**) Dose–response relationship between dietary VD intake and the risk of low cognitive performance (Animal Fluency test); (**b**) Dose–response relationship between dietary VD intake and the risk of low cognitive performance (DSST); (**c**) Dose–response relationship between serum total 25(OH)D concentration and the risk of low cognitive performance (DSST); (**d**) Dose–response relationship between serum 25(OH)D_3_ concentration and the risk of low cognitive performance (DSST). The solid line represents the OR values and dashed lines represent the 95% confidence intervals.

As shown in Figure 2, L-shaped dose–response relationships were found between dietary VD intake and the risk of low cognitive performance (the Animal Fluency test, *P*_for nonlinearity_ = 0.426; DSST, *P*_for nonlinearity_ = 0.239). Additionally, L-shaped dose–response relationships were also found in between serum total 25(OH)D and the risk of low cognitive performance (DSST, *P*_for nonlinearity_ = 0.697), as well as found in between serum 25(OH)D_3_ and the risk of low cognitive performance (DSST, *P*_for nonlinearity_ = 0.409). For the Animal Fluency test, when dietary VD intake was higher than 20 μg/d, the risk of low cognitive performance began to decrease (Figure 2a); for the DSST, the risk of low cognitive performance began to decrease when dietary VD intake was higher than 4 μg/d (Figure 2b). When the serum total 25(OH)D concentration was higher than 87 nmol/L, the serum total 25(OH)D was statistically significantly associated with the decreased risk of low cognitive performance in DSST (Figure 2c). The serum 25(OH)D_3_ was also associated with a decreased risk of low cognitive performance assessed by DSST when 25(OH)D_3_ concentration was higher than 44 nmol/L (Figure 2d).

## 4. Discussion

In this study, we investigated the associations between dietary VD intake and serum 25(OH)D with cognitive performance in older adults. The study found that the dietary VD intake, serum total 25(OH)D, and 25(OH)D_3_ concentration were negatively associated with low cognitive performance risk, and linear L-shaped dose–response relationships between them were identified. In stratified analysis by gender, the associations between dietary VD intake, serum total 25(OH)D, and cognitive performance were not different between genders.

Recent observational studies have researched the relationship between dietary VD intake and cognitive performance [11,18,38,39,40]. The study of Przybelski et al. showed that elderly adults with higher levels of VD intake had better cognitive performance [40]. Additionally, other studies came to the same conclusion, which was consistent with our study findings [18,38], while the study of Elske et al. showed that the effect of dietary VD intake on cognitive performance was non-significant. [11] Our results were inconsistent with this study and the reason may be the use of different populations. Elske et al. included 127 Dutch older adults over 65 years, but our study included 2524 older adults over 60 years. In addition, in our study, dietary vitamin D intake was grouped by RNIs (5.00 μg/d) provided by FAO/WHO, and the results show that RNIs were also preferable for protecting cognitive performance.

For serum 25(OH)D, some studies supported our view [17,41,42,43]. The study of Littlejohns et al. found a negative correlation between the incidence of Alzheimer’s disease (AD) and serum total 25(OH)D concentration [41]. Additionally, another French cohort study showed that higher level of serum total 25(OH)D could delay the development of cognitive decline and dementia in older adults [42]. The relationship between serum 25(OH)D_2_, 25(OH)D_3_ and cognition has not been explored in past studies. In our study, we found that higher 25(OH)D_3_ concentrations had a protective effect on cognitive performance, while the association between 25(OH)D_2_ and cognitive performance was not significant. Meanwhile, a study involved patients with AD or mild cognitive impairment (MCI) found that 25(OH)D_3_ reduced the risk of MCI and AD [44]. It is important to further explore the role of 25(OH)D_3_ in cognitive performance.

At the same time, in our study, we found that the associations between dietary VD intake, serum total 25(OH)D, and cognitive performance remained unchanged between genders. The research of Morello et al. supported our point [45]. In addition, some studies only focused on women [10,46,47], and few studies explored the effect of serum 25(OH)D on cognition in males; therefore, the relationship between 25(OH)D and cognition in males should not be ignored in future research.

To our knowledge, no studies have explored the dose–response relationships between dietary VD intake, serum 25(OH)D, and cognitive performance. In our study, we found a linear L-shaped dose–response relationship between dietary VD intake, serum total 25(OH)D, serum 25(OH)D_3_, and cognition. Referable values of serum total 25(OH)D, 25(OH)D_3_ concentration, and dietary VD intake were proposed to protect cognitive performance, which need to be researched by further studies.

Increasingly, recent studies have focused on the influence of VD on cognitive performance. Several studies have shown that VD affects cognitive performance by affecting cell differentiation, neurotransmitter synthesis, the expression of genes and proteins involved in neural structure, and so on [48]. These action mechanisms emphasize the important role of VD in brain function, so we think that VD may be an important nutrient for maintaining better cognitive performance and preventing cognitive decline in the elderly.

There are several advantages present in the study. First of all, we explored both the dose–response relationships of dietary VD intake, serum total 25(OH)D, 25(OH)D_3_, and 25(OH)D_2_ concentrations with cognitive performance, respectively. Referable cutoff values of serum 25(OH)D concentration and dietary VD intake were also provided. In addition, when we explored the relationship between dietary VD intake and cognitive performance, we not only adopted the three-digit grouping, but also referred to RNIs for grouping. Next, we explored the associations between 25(OH)D_3_, 25(OH)D_2_ and cognition, respectively, and found that 25(OH)D_3_ were related to cognitive performance, while 25(OH)D_2_ was not. Finally, we explored gender differences in dietary VD intake, serum total 25(OH)D concentration, and cognitive performance, respectively.

However, there are some limitations in the study. First of all, the study was a cross-sectional study and could not determine the cause and effect. Secondly, the data of dietary VD intake obtained through 24 h dietary recall could not accurately judge individual dietary intake, and there was recall bias. In the next place, comparing the included with excluded populations, there were no differences in gender (serum: *p* = 0.199; diet: *p* = 0.661), but differences in age, race, education level, marital status, and income level. Although we conducted a weighted analysis, extrapolation still needs to be approached with caution. Finally, after adjusting for all covariates, dietary VD intake and serum 25(OH)D were only associated with cognitive performance assessed by DSST, not with all cognitive dimensions.

## 5. Conclusions

In the study, the associations between dietary VD intake, serum total 25(OH)D, 25(OH)D_2_, 25(OH)D_3_, and cognitive performance were investigated separately and positive associations between dietary VD intake, serum total 25(OH)D, 25(OH)D_3_ with cognitive performance were found. In addition, L-shaped dose–response relationships of them with cognitive performance were found. Future studies should delve into the relationships between them and mechanisms of their impact on cognition, respectively.

## Figures and Tables

**Table 1:** The cognitive performance cutoff points of CERAD test, Animal Fluency test, and DSST score adjusted according to age (≥60 years and ≥70 years).

	CERAD Test Score	Animal Fluency Test Score	Digit Symbol Test Score
Dietary VD intake (μg/d)			
≥60 years	23	14	38
≥70 years	19	12	31
Serum 25-hydroxyvitamin D (nmol/L)			
≥60 years	22	14	37
≥70 years	19	12	30

**Table 2:** Characteristics of the dietary VD and cognition study population, National Health and Nutrition Examination Survey (NHANES) 2011–2014 (N = 2524).

		CERAD Test			Animal Fluency Test			Digit Symbol Test		
	Number of Subjects (N)	Normal Cognitive Performance	Low Cognitive Performance	*p*-Value	Normal Cognitive Performance	Low Cognitive Performance	*p*-Value	Normal Cognitive Performance	Low Cognitive Performance	*p*-Value
Number of subjects (%)		1806(71.6)	718(28.4)		1792(71.0)	731(29.0)		1867(74.0)	647(26.0)	
Age(%) ^1^	2524			0.028			0.415			0.993
≥60 years		954(52.8)	414(57.7)		962(53.7)	406(55.5)		1012(54.2)	356(54.2)	
≥70 years		852(47.2)	304(42.3)		830(46.3)	326(44.5)		855(45.8)	301(45.8)	
Gender(%) ^1^	2524			<0.01			0.664			<0.01
Male		785(43.5)	432(60.2)		869(48.5)	348(47.5)		847(45.4)	370(56.3)	
Female		1021(56.5)	286(39.8)		923(51.5)	384(52.5)		1020(54.6)	287(43.7)	
Race(%) ^1^	2524			<0.01			<0.01			<0.01
Mexican American		129(7.1)	82(11.4)		149(8.3)	62(8.5)		119(6.4)	92(14.0)	
Other Hispanic		141(7.8)	103(14.3)		151(8.4)	93(12.7)		120(6.4)	124(18.9)	
Non-Hispanic White		971(53.8)	298(41.5)		1033(57.6)	236(32.2)		1094(58.6)	175(26.6)	
Non-Hispanic Black		401(22.3)	192(26.7)		334(18.6)	260(35.5)		350(18.7)	244(37.1)	
Other races		163(9.0)	43(6.0)		125(7.0)	81(11.1)		184(9.9)	22(3.3)	
Educational level (%) ^1^	2522			<0.01			<0.01			<0.01
Below high school		382(19.0)	304(42.2)		369(19.3)	317(38.6)		276(13.7)	410(56.9)	
High school		475(23.6)	175(24.3)		435(22.7)	215(26.2)		489(24.3)	161(22.3)	
Above high school		1158(57.5)	241(33.5)		1110(58.0)	289(35.2)		1249(62.0)	150(20.8)	
Marital status (%) ^1^	2521			0.883			0.027			<0.01
Married/living with partner		1060(58.8)	419(58.4)		1075(60.1)	404(55.3)		1150(61.7)	329(50.2)	
Widowers/divorced/separated/never married		744(41.2)	298(42.6)		715(39.9)	317(44.7)		715(38.3)	327(49.8)	
Poverty-income ratio (%) ^1^	2333			<0.01			<0.01			<0.01
≤1.00		228(13.6)	145(22.0)		219(13.1)	154(23.2)		192(11.1)	181(30.4)	
≥1.00		1446(86.4)	514(78.0)		1450(86.9)	510(76.8)		1545(88.9)	415(69.6)	
Body mass index (%) ^1^	2492			0.163			0.662			0.886
<25 kg/m^2^		466(26.1)	195(27.6)		465(26.6)	196(27.5)		491(26.5)	170(26.7)	
<30 kg/m^2^		607(34.0)	259(36.6)		627(35.3)	239(33.5)		650(35.0)	216(34.0)	
≥30 kg/m^2^		712(39.9)	253(35.8)		686(38.6)	279(39.1)		715(38.5)	250(39.3)	
Physical activity level(%) ^1^	2524			<0.01			<0.01			<0.01
Moderate and high		803(44.5)	264(36.8)		825(46.0)	242(33.1)		875(46.9)	192(29.2)	
Low		1003(55.5)	454(63.2)		968(54.0)	490(66.9)		992(53.1)	465(70.8)	
Season of exam (%) ^1^	2524			0.545			0.832			0.023
November-April		796(44.1)	326(45.4)		799(44.6)	323(44.1)		805(43.1)	317(48.2)	
May-October		1010(55.9)	392(54.6)		993(55.4)	409(55.9)		1128(56.0)	340(51.8)	
Smoking status (%) ^1^	2522	907(50.3)	368(51.3)	0.658	915(51.1)	360(49.2)	0.377	1329(71.5)	401(62.0)	0.796
Hypertension (%) ^1^	2522	1248(69.1)	514(71.7)	0.209	1212(67.7)	550(75.1)	<0.01	1267(67.9)	495(75.3)	<0.01
Diabetes (%) ^1^	2524	467(25.9)	232(32.3)	0.001	440(24.6)	259(35.4)	<0.01	443(23.7)	256(39.0)	<0.01
Stroke (%) ^1^	2519	107(5.9)	62(8.6)	0.015	100(5.6)	69(9.5)	<0.01	94(5.0)	75(11.4)	<0.01
Had at least 12 alcohol drinks/year (%) ^1^	2506	1244(69.1)	486(68.7)	0.842	1271(71.3)	459(63.4)	<0.01	1329(71.5)	401(62.0)	<0.01
Total energy intake (kcal/day) ^2^	2524	1845.71(672.87)	1761.34(685.91)	0.001	1885.32(676.43)	1666.00(655.11)	<0.01	1875.86(651.83)	1667.83(724.50)	<0.01
Daily dietary VD intake (μg/d) ^2^	2524	25.14(47.43)	19.04(58.55)	<0.01	24.95(49.71)	19.60(53.54)	<0.01	26.49(57.00)	14.60(24.79)	<0.01

Data is number of subjects (percentage) or medians (interquartile ranges); ^1^ Chi-square was used to compare the percentage between participants with and without low cognitive performance; ^2^ Mann–Whitney U test was applied to compare the median values between participants with and without low cognitive performance.

**Table 3:** Characteristics of the serum 25(OH)D and cognition study population, National Health and Nutrition Examination Survey (NHANES) 2011–2014 (N = 2735).

		CERAD Test			Animal Fluency Test			Digit Symbol Test		
	Number of Subjects (N)	Normal Cognitive Performance	Low Cognitive Performance	*p*-Value	Normal Cognitive Performance	Low Cognitive Performance	*p*-Value	Normal Cognitive Performance	Low Cognitive Performance	*p*-Value
Number of subjects (%)		2015(73.7)	720(26.3)		1914(70.0)	821(30.0)		2014(73.6)	721(26.4)	
Age (%) ^1^	2735			0.258			0.449			0.963
≥60 years		1110(55.1)	379(52.6)		1033(54.0)	456(55.0)		1097(54.5)	392(54.4)	
≥70 years		905(44.9)	341(47.4)		881(46.0)	365(44.5)		917(45.5)	329(45.6)	
Gender(%) ^1^	2735			<0.01			0.460			<0.01
Male		911(45.2)	431(59.9)		948(49.5)	394(48.0)		941(46.7)	401(55.6)	
Female		1104(54.8)	289(40.1)		966(50.5)	427(52.0)		1073(53.3)	320(44.4)	
Race(%) ^1^	2735			<0.01			<0.01			<0.01
Mexican American		160(7.9)	81(11.3)		169(8.8)	72(8.8)		137(6.8)	104(14.4)	
Other Hispanic		177(8.8)	102(14.2)		173(9.0)	106(12.9)		138(6.9)	141(19.6)	
Non-Hispanic White		1032(51.2)	190(40.3)		1057(55.2)	265(32.3)		1141(56.7)	181(25.1)	
Non-Hispanic Black		441(21.9)	189(26.3)		359(18.8)	271(33.0)		380(18.9)	250(34.7)	
Other races		205(10.2)	58(8.1)		156(8.2)	107(13.0)		218(10.8)	45(6.2)	
Educational level (%) ^1^	2735			<0.01			<0.01			<0.01
Below high school		382(19.0)	304(42.2)		369(19.3)	317(38.6)		276(13.7)	410(56.9)	
High school		475(23.6)	175(24.3)		435(22.7)	215(26.2)		489(24.3)	161(22.3)	
Above high school		1158(57.5)	241(33.5)		1110(58.0)	289(35.2)		1249(62.0)	150(20.8)	
Marital status (%) ^1^	2733			0.089			0.020			<0.01
Married/living with partner		1189(59.1)	399(55.4)		11139(59.5)	449(54.8)		1226(60.9)	392(50.3)	
widowers/divorced/separated/never married		824(40.9)	321(44.6)		774(40.5)	371(45.2)		78(39.1)	358(49.7)	
Poverty-income ratio (%) ^1^	2509			0.199			0.928			0.572
≤1.00		318(17.1)	96(14.9)		292(16.5)	122(16.4)		312(16.7)	102(15.8)	
≥1.00		1546(82.9)	549(85.1)		1473(83.5)	622(83.6)		1551(83.3)	544(84.2)	
Body mass index (%) ^1^	2697			0.178			0.449			0.675
<25 kg/m^2^		535(26.9)	205(29.0)		508(26.8)	232(29.0)		543(27.2)	197(28.2)	
<30 kg/m^2^		699(35.1)	360(36.8)		685(36.1)	274(31.1)		706(35.3)	253(36.2)	
≥30 kg/m^2^		757(38.0)	241(34.1)		705(37.1)	293(36.7)		749(37.5)	249(35.6)	
Physical activity level (%) ^1^	2735			<0.01			<0.01			<0.01
Moderate and high		892(44.3)	258(35.8)		889(46.4)	261(31.8)		946(47.0)	204(28.3)	
Low		1123(55.7)	462(64.2)		1025(53.6)	560(68.2)		1068(53.0)	517(71.7)	
Season of exam (%) ^1^	2735			0.877			0.859			0.011
November-April		914(45.4)	329(45.7)		872(45.6)	371(45.2)		886(44.0)	357(49.5)	
May-October		1101(54.6)	391(54.3)		1042(54.4)	450(54.8)		1128(56.0)	364(50.5)	
Smoking status (%) ^1^	2733	1013(50.3)	369(51.3)	0.669	969(50.7)	413(50.3)	0.857	1005(50.0)	377(52.3)	0.281
Hypertension (%)^1^	2733	1381(68.6)	514(71.5)	0.145	1283(67.1)	612(74.5)	<0.01	1357(67.4)	538(74.6)	<0.01
Diabetes (%) ^1^	2735	529(26.3)	230(31.9)	0.003	473(24.7)	286(34.8)	<0.01	480(23.8)	279(38.7)	<0.01
Stroke (%)	2730	115(5.7)	72(10.0)	<0.01	104(5.4)	83(10.1)	<0.01	105(5.2)	82(11.4)	<0.01
Had at least 12 alcohol drinks/year (%) ^1^	2689	1376(69.2)	466(66.6)	0.201	1339(70.9)	503(63.8)	<0.01	1413(70.9)	429(61.5)	<0.01
Serum total 25(OH)D (nmol/L) ^2^	2735	78.17(32.02)	72.55(30.20)	<0.01	78.3(31.1)	72.68(32.84)	<0.01	79.06(31.33)	69.72(31.78)	<0.01
Serum 25(OH)D_3_ (nmol/L) ^2^	2735	71.38(32.31)	65.32(29.33)	<0.01	71.90(31.55)	64.91(31.37)	<0.01	72.43(31.62)	62.04(30.73)	<0.01
Serum 25(OH)D_2_ (nmol/L) ^1^	2735			0.076			0.159			0.45
≤1.45		1512(75.0)	516(71,7)		1434(74.9)	594(72.4)		1501(74.5)	527(73.1)	
>1.45		503(25.0)	204(28.3)		480(25.1)	227(27.6)		513(25.5)	194(26.9)	

Data are number of subjects (percentage) or medians (interquartile ranges); ^1^ Chi-square was used to compare the percentage between participants with and without low cognitive performance; ^2^ Mann–Whitney U test was applied to compare the median values between participants with and without low cognitive performance.

**Table 4:** Weighted ORs (95%CI) for scores on the Consortium to CERAD test, Animal Fluency test, DSST across dietary VD intake, NHANES 2011–2014 (N = 2524).

		**Dietary VD Intake (μg/d)**			**Dietary VD Intake (μg/d)**	
		**≤5.00**	**≤20.75**	**>20.75**	**≤5.00**	**>5.00**
CREAD Test	Case/Participants	265/798	268/888	185/838	265/798	453/1726
	Crude	1.00 (Ref.)	1.02 (0.80–1.30)	0.69 (0.51–0.92) *	1.00 (Ref.)	0.84 (0.67–1.04)
	Model 1	1.00 (Ref.)	0.96 (0.75–1.24)	0.70 (0.51–0.96) *	1.00 (Ref.)	0.82 (0.65–1.05)
	Model 2	1.00 (Ref.)	0.97 (0.72–1.29)	0.83 (0.58–1.19)	1.00 (Ref.)	0.90 (0.69–1.17)
Animal Fluency Test	Case/Participants	284/798	260/888	188/838	284/798	448/1726
	Crude	1.00 (Ref.)	0.69 (0.49–0.97) *	0.43 (0.34–0.55) *	1.00 (Ref.)	0.55 (0.43–0.70) *
	Model 1	1.00 (Ref.)	0.67 (0.48–0.93) *	0.41 (0.31–0.53) *	1.00 (Ref.)	0.53 (0.41–0.67) *
	Model 2	1.00 (Ref.)	0.70 (0.43–1.13)	0.51 (0.36–0.72) *	1.00 (Ref.)	0.60 (0.41–0.89) *
Digit Symbol Test	Case/Participants	277/798	247/888	133/838	277/798	380/1726
	Crude	1.00 (Ref.)	0.82 (0.55–1.20)	0.36 (0.26–0.51) *	1.00 (Ref.)	0.56 (0.41–0.77) *
	Model 1	1.00 (Ref.)	0.77 (0.53–1.14)	0.34 (0.25–0.46) *	1.00 (Ref.)	0.53 (0.39–0.73) *
	Model 2	1.00 (Ref.)	0.72 (0.45–1.15)	0.45 (0.31–0.66) *	1.00 (Ref.)	0.59 (0.40–0.86) *

Binary logistic regression analyses were used to calculate weighted OR values. Reference (Ref.); model 1 adjusted for age and gender; model 2 adjusted for age and gender, race, educational level, marital status, income, body mass index (BMI), season of exam, physical activity level, drinking status, smoking status, hypertension, diabetes, and stroke. Energy was adjusted, when we studied the intake of dietary VD. * *p*-value ≤ 0.05.

**Table 5:** Weighted ORs (95% CI) for score on CERAD test, Animal Fluency test and DSST across dietary VD intake and serum total 25(OH)D, stratified by gender, NHANES 2011–2014.

	CREAD Test				Animal Fluency Test				Digit Symbol Test			
	Case/Participants	Crude	Model 1	Model 2	Case/Participants	Crude	Model 1	Model 2	Case/Participants	Crude	Model 1	Model 2
Dietary VD intake (μg/d)												
male	432/1217				348/1217				370/1217			
≤5.00		1.00 (Ref.)	1.00 (Ref.)	1.00 (Ref.)		1.00 (Ref.)	1.00 (Ref.)	1.00 (Ref.)		1.00 (Ref.)	1.00 (Ref.)	1.00 (Ref.)
≤20.75		1.04 (0.66–1.64)	1.04 (0.66–1.64)	1.09 (0.68–1.75)		0.65 (0.35–1.20)	0.64 (0.35–1.17)	0.73 (0.37–1.44)		0.95 (0.58–1.54)	0.93 (0.56–1.53)	0.90 (0.55–1.47)
>20.75		0.79 (0.46–1.36)	0.79 (0.45–1.40)	0.86 (0.44–1.68)		0.34 (0.20–0.57)	0.31 (0.19–0.55)	0.38 (0.22–0.67)		0.36 (0.23–0.58)	0.34 (0.22–0.55)	0.46 (0.26–0.82)
female	286/1217				184/1217				287/1217			
≤5.00		1.00 (Ref.)	1.00 (Ref.)	1.00 (Ref.)		1.00 (Ref.)	1.00 (Ref.)	1.00 (Ref.)		1.00 (Ref.)	1.00 (Ref.)	1.00 (Ref.)
≤20.75		0.90 (0.61–1.33)	0.88 (0.60–1.27)	0.80 (0.49–1.32)		0.73 (0.50–1.06)	0.70 (0.47–1.03)	0.69 (0.46–1.05)		0.66 (0.41–1.07)	0.62 (0.39–1.00)	0.57 (0.26–1.25)
>20.75		0.66 (0.42–1.02) *	0.62 (0.42–0.93) *	0.81 (0.53–1.23)		0.51 (0.34–0.77) *	0.48 (0.31–0.72) *	0.68 (0.43–1.07)		0.36 (0.23–0.57) *	0.33 (0.21–0.50) *	0.50 (0.30–0.83) *
Dietary VD intake (μg/d)												
male	432/1217				348/1217				370/1217			
≤5.00		1.00 (Ref.)	1.00 (Ref.)	1.00 (Ref.)		1.00 (Ref.)	1.00 (Ref.)	1.00 (Ref.)		1.00 (Ref.)	1.00 (Ref.)	1.00 (Ref.)
>5.00		0.93 (0.62–1.38)	0.93 (0.62–1.40)	1.00 (0.64–1.56)		0.51 (0.30–0.85) *	0.49 (0.30–0.83) *	0.58 (0.33–1.04)		0.68 (0.44–1.03)	0.65 (0.42–1.02)	0.72 (0.47–1.10)
female	286/1217				184/1217				287/1217			
≤5.00	432/1217	1.00 (Ref.)	1.00 (Ref.)	1.00 (Ref.)		1.00 (Ref.)	1.00 (Ref.)	1.00 (Ref.)		1.00 (Ref.)	1.00 (Ref.)	1.00 (Ref.)
>5.00		0.75 (0.52–1.07)	0.72 (0.52–0.99) *	0.81 (0.54–1.19)		0.59 (0.41–0.85) *	0.56 (0.38–0.81) *	0.68 (0.47–1.00) *		0.47 (0.32–0.69) *	0.44 (0.30–0.63) *	0.53 (0.31–0.93) *
Total 25-Hydroxyvitamin D (nmol/L)												
male	431/1342				394/1342				401/1342			
≤61.41		1.00 (Ref.)	1.00 (Ref.)	1.00 (Ref.)		1.00 (Ref.)	1.00 (Ref.)	1.00 (Ref.)		1.00 (Ref.)	1.00 (Ref.)	1.00 (Ref.)
≤86.30		0.83 (0.59–1.17)	0.84 (0.60–1.18)	0.99 (0.65–1.51)		0.56 (0.39–0.85) *	0.57 (0.38–0.85) *	0.84 (0.54–1.30)		0.44 (0.28–0.68) *	0.44 (0.29–0.68) *	0.84 (0.44–1.61)
>86.30		0.63 (0.43–0.93) *	0.61 (0.41–0.89) *	0.77 (0.46–1.31)		0.46 (0.25–0.84) *	0.44 (0.25–0.79) *	0.65 (0.35–1.23)		0.35 (0.20–0.63) *	0.35 (0.20–0.61) *	0.67 (0.30–1.48)
female	289/1393				427/1393				320/1393			
≤61.41		1.00 (Ref.)	1.00 (Ref.)	1.00 (Ref.)		1.00 (Ref.)	1.00 (Ref.)	1.00 (Ref.)		1.00 (Ref.)	1.00 (Ref.)	1.00 (Ref.)
≤86.30		1.09 (0.75–1.58)	1.04 (0.70–1.53)	1.01 (0.67–1.54)		1.03 (0.69–1.55)	0.99 (0.65–1.51)	1.49 (0.93–2.41)		0.84 (0.55–1.29)	0.78 (0.50–1.23)	1.04 (0.61–1.78)
>86.30		0.82 (0.58–1.15)	0.73 (0.50–1.06)	0.75 (0.47–1.20)		0.83 (0.58–1.19)	0.76 (0.53–1.07)	1.30 (0.92–1.83)		0.56 (0.41–0.76) *	0.47 (0.34–0.66) *	0.68 (0.42–1.09)

Binary logistic regression analyses were used to calculate weighted OR values. Reference (Ref.); model 1 adjusted for age and gender; model 2 adjusted for age and gender, race, educational level, marital status, income, body mass index (BMI), season of exam, physical activity level, drinking status, smoking status, hypertension, diabetes, and stroke. Energy was adjusted when we studied the intake of dietary VD. * *p*-value ≤ 0.05.

**Table 6:** Weighted ORs (95%CI) for scores on the Consortium to CERAD test, Animal Fluency test, DSST across quartiles of serum total 25(OH)D, 25(OH)D_2_ and 25(OH)D_3_, NHANES 2011–2014 (N = 2735).

	CERAD Test				Animal Fluency Test				Digit Symbol Test			
	Case/Participants	Crude	Model 1	Model 2	Case/Participants	Crude	Model 1	Model 2	Case/Participants	Crude	Model 1	Model 2
Total 25-Hydroxyvitamin D (nmol/L)												
≤61.41	274/911	1.00 (Ref.)	1.00 (Ref.)	1.00 (Ref.)	326/911	1.00 (Ref.)	1.00 (Ref.)	1.00 (Ref.)	307/911	1.00 (Ref.)	1.00 (Ref.)	1.00 (Ref.)
≤86.30	252/915	0.96 (0.77–1.20)	0.94 (0.75–1.15)	1.02 (0.77–1.34)	259/915	0.75 (0.59–0.96) *	0.75 (0.58–0.97) *	1.12 (0.83–1.51)	237/915	0.60 (0.44–0.81) *	0.58 (0.43–0.80) *	0.93 (0.61–1.42)
>86.30	194/909	0.68 (0.53–0.87) *	0.67 (0.52–0.86) *	0.77 (0.55–1.08)	236/909	0.64 (0.47–0.88) *	0.59 (0.44–0.81) *	0.95 (0.70–1.28)	177/909	0.44 (0.34–0.55) *	0.41 (0.32–0.52) *	0.68 (0.47–0.97) *
25-Hydroxyvitamin D_3_ (nmol/L)												
≤55.14	264/911	1.00 (Ref.)	1.00 (Ref.)	1.00 (Ref.)	334/911	1.00 (Ref.)	1.00 (Ref.)	1.00 (Ref.)	316/911	1.00 (Ref.)	1.00 (Ref.)	1.00 (Ref.)
≤80.63	266/913	1.16 (0.85–1.59)	1.10 (0.81–1.48)	1.23 (0.86–1.77)	259/913	0.74 (0.56–0.99) *	0.74 (0.56–0.98) *	1.10 (0.92–1.46)	238/913	0.61 (0.46–0.81) *	0.58 (0.43–0.80) *	0.92 (0.64–1.33)
>80.63	190/911	0.74 (0.53–1.03)	0.72 (0.51–1.02)	0.92 (0.58–1.45)	228/911	0.61 (0.47–0.99) *	0.57 (0.44–0.75) *	0.98 (0.75–1.29)	167/911	0.36 (0.29–0.46) *	0.41 (0.32–0.52) *	0.62 (0.44–0.86) *
25-Hydroxyvitamin D_2_ (nmol/L)												
≤1.45	516/2028	1.00 (Ref.)	1.00 (Ref.)	1.00 (Ref.)	594/2028	1.00 (Ref.)	1.00 (Ref.)	1.00 (Ref.)	527/2028	1.00 (Ref.)	1.00 (Ref.)	1.00 (Ref.)
>1.45	204/707	1.27 (0.92–1.77)	1.30 (0.93–1.81)	1.47 (0.98–2.22)	227/707	0.99 (0.74–1.33)	0.97 (0.72–1.29)	1.05 (0.74–1.47)	194/707	0.95 (00.73–1.25)	0.94 (0.70–1.26)	1.00 (0.68–1.49)

Binary logistic regression analyses were used to calculate weighted OR values. Reference (Ref.); model 1 adjusted for age and gender; model 2 adjusted for age and gender, race, educational level, marital status, income, body mass index (BMI), season of exam, physical activity level, drinking status, smoking status, hypertension, diabetes, and stroke. Energy was adjusted when we studied the intake of dietary VD. * *p*-value ≤ 0.05.

## Data Availability

The data that support the findings of this study are openly available vis this link: http://www.cdc.gov/nchs/nhanes.htm(accessed on 28 June 2021).
